# Growth Inhibition and Apoptosis Induced by Osthole, A Natural Coumarin, in Hepatocellular Carcinoma

**DOI:** 10.1371/journal.pone.0037865

**Published:** 2012-05-25

**Authors:** Lurong Zhang, Guorong Jiang, Fei Yao, Yan He, Guoqiang Liang, Yinsheng Zhang, Bo Hu, Yan Wu, Yunsen Li, Haiyan Liu

**Affiliations:** 1 Laboratory of Cellular and Molecular Tumor Immunology, Jiangsu Key Laboratory of Infection and Immunity, Institutes of Biology and Medical Sciences, Soochow University, Suzhou, People's Republic of China; 2 Suzhou Hospital of Traditional Chinese Medicine, Suzhou, People's Republic of China; 3 Cyrus Tang Hematology Center, Department of Hematology, Jiangsu Institute of Hematology, the First Affiliated Hospital of Soochow University, Suzhou, People's Republic of China; Royal College of Surgeons, Ireland

## Abstract

**Background:**

Hepatocellular carcinoma (HCC) is one of the most commonly diagnosed tumors worldwide and is known to be resistant to conventional chemotherapy. New therapeutic strategies are urgently needed for treating HCC. Osthole, a natural coumarin derivative, has been shown to have anti-tumor activity. However, the effects of osthole on HCC have not yet been reported.

**Methods and Findings:**

HCC cell lines were treated with osthole at various concentrations for 24, 48 and 72 hours. The proliferations of the HCC cells were measured by MTT assays. Cell cycle distribution and apoptosis were determined by flow cytometry. HCC tumor models were established in mice by subcutaneously injection of SMMC-7721 or Hepa1-6 cells and the effect of osthole on tumor growths *in vivo* and the drug toxicity were studied. NF-κB activity after osthole treatment was determined by electrophoretic mobility shift assays and the expression of caspase-3 was measured by western blotting. The expression levels of other apoptosis-related genes were also determined by real-time PCR (PCR array) assays. Osthole displayed a dose- and time-dependent inhibition of the HCC cell proliferations *in vitro*. It also induced apoptosis and caused cell accumulation in G2 phase. Osthole could significantly suppress HCC tumor growth *in vivo* with no toxicity at the dose we used. NF-κB activity was significantly suppressed by osthole at the dose- and time-dependent manner. The cleaved caspase-3 was also increased by osthole treatment. The expression levels of some apoptosis-related genes that belong to TNF ligand family, TNF receptor family, Bcl-2 family, caspase family, TRAF family, death domain family, CIDE domain and death effector domain family and CARD family were all increased with osthole treatment.

**Conclusion:**

Osthole could significantly inhibit HCC growth *in vitro* and *in vivo* through cell cycle arrest and inducing apoptosis by suppressing NF-κB activity and promoting the expressions of apoptosis-related genes.

## Introduction

Hepatocellular carcinoma (HCC) is the most common primary malignant tumor of the liver and accounts for about 5.6% of all tumors [1]. Because of its poor prognosis, it is the third most common cause of cancer mortality [2], [3], [4]. HCC is highly aggressive and resistant to conventional therapies such as radiotherapy and chemotherapy [5], [6], [7]. Therefore, more effective therapeutic agents for treating HCC are desirable.

Previous studies have shown that some natural chemopreventive agents can induce apoptosis of tumor cells and inhibit tumor growth, including HCC, both in *vitro* and in *vivo* [8], [9], [10], [11], [12], [13], [14]. Because of their selectivity of killing tumor cells and minimal toxicity comparing with conventional chemotherapies, they are becoming promising approaches for tumor treatments. Coumarins and its known metabolite 7-hydroxy-coumarin have been shown to have growth suppressive effect on many cancer cell lines, such as colon-carcinoma cell lines, hepatocellular carcinoma cell lines, leukemia cell lines, melanoma cell lines, renal cell carcinoma cell lines and non-small cell lung carcinoma cell lines [15], [16], [17]. Auraptene, one of the coumarins, has been shown to be effective in inhibiting the development of esophageal tumors and colitis-related colon cancers in animal models [18], [19]. Coumarin has also been used in a clinical trial to prevent disease recurrence in melanoma patients [20]. They can affect multiple signaling pathways, such as ERK/MAPK and PI3K/Akt pathways, which play important roles in carcinogenesis [17], [21], [22], [23], [24].

Osthole, 7-methoxy-8-(3-methyl-2-butenyl) coumarin, a bioactive simple coumarin derivative extracted from many medicinal plants such as *Cnidium monnieri* (L.) Cusson, has long been used in traditional Chinese medicine for the treatment of eczema, cutaneous pruritus, trichomonas vaginalis infection, and sexual dysfunction. Recent studies have revealed that osthole has comprehensive and wider applications with anti-inflammatory, anti-osteoporotic, anti-bacterial, and anti-allergic effects [25], [26], 27,28,29. Furthermore, accumulating evidence indicates that osthole possesses anti-tumor effects by inhibiting tumor cell growth and inducing apoptosis [30], [31], [32], [33]. It has been reported recently that osthole was able to inhibit the migration and invasion of breast cancer cells [34]. However, the effects of osthole on HCC remain unknown.

The molecular mechanism of osthole’s anti-tumor effect was not yet clearly known. It has been shown that osthole-induced G2/M arrest and apoptosis in lung cancer A549 cells were associated with the inhibition of Cyclin B1, p-Cdc2 and p-Akt expressions and up-regulation of the Bax/Bcl-2 ratios [33]. Osthole could also down-regulate fatty acid synthase (FASN) expression and induce apoptosis in HER2-overexpressing breast cancer cells through inhibiting the phosphorylation of Akt and mTOR [35]. Furthermore, osthole has been shown to effectively inhibit MMP-2 promoter and enzyme activity, which might be one of the causes that lead to the inhibition of migration and invasion of breast cancer cells by osthole [34]. More studies are needed to fully address the molecular mechanisms of the anti-tumor effects of osthole.

In the present study, osthole was found to inhibit proliferation and induce apoptosis of HCC cell lines. Osthole treatment significantly suppressed the tumor growth in nude mice and C57/BL6 mice. Our results also suggested that osthole could inhibit NF-κB activity and up-regulate the expression levels of apoptosis-related genes. Therefore, osthole could be a good compound for developing anticancer agents for HCC.

## Materials and Methods

### Cell Lines and Culture Conditions

Human HCC cell line HepG2 and murine HCC cell line Hepa1-6 were gifts from Dr. Limin Zheng (School of Life Sciences, Sun Yat-sen University). Human HCC cell line HepG2 was originally purchased from ATCC (Manassas, VA). Hepa1-6 was originally purchased from Cell Bank, Chinese Academy of Sciences (Shanghai, China). Human HCC cell lines SMMC-7721 and SK-HP-1 were purchased from Cell Bank, Chinese Academy of Sciences (Shanghai, China). Cells were maintained in Dulbecco’s modified Eagle’s medium (DMEM) with high glucose (Gibco, Grand Island, NY) supplemented with 10% heat-inactivated fetal bovine serum (Gibco, Grand Island, NY) at 37°C in a humidified atmosphere containing 5% CO_2_.

**Figure 1:**
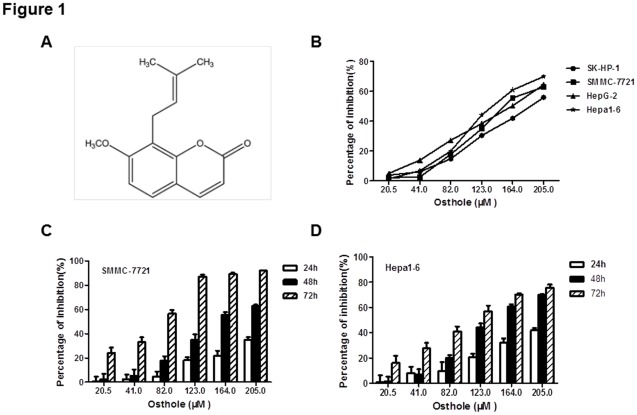
Osthole Inhibited Cell Proliferation of HCC Cell Lines. (A) Chemical Structure of osthole (B) Viability of SK-HP-1, SMMC-7721, HepG-2 and Hepa1-6 cells treated with osthole. MTT assay was performed to measure cell growth inhibition rate at 48 h after osthole treatment. (C)(D) Viability of SMMC-7721 and Hepa1-6 cells treated with osthole. MTT assay was performed to measure cell growth inhibition rate at 24 h, 48 h and 72 h after osthole treatment. Data shown were representatives of three experiments.

### Antibodies and Reagents

Antibodies against β-actin and caspase-3 were purchased from Cell Signaling (Boston, MA). Osthole (molecular weight 244.29) was purchased from National Institutes for Food and Drug Control (Beijing, China). Nuclear and Cytoplasmic Protein Extraction Kit and BCA Protein Assay Kit were purchased from Beyotime (Jiangsu, China). Annexin V-FITC and PI double staining kit were purchased from Key Gene (Jiangsu, China). NF-κB EMSA Kit was purchased from Viagene Biotech (Tampa, FL). RT^2^ Profiler PCR Arrays was purchased from SABiosciences Corporation (Frederick, MD). Osthole was purchased from National Institutes for Food and Drug Control (Beijing, China). It is over 99% pure determined by HPLC. A stock solution of osthole (205.0 µM) was prepared by dissolving in DMEM with 0.25% ethanol and 0.25% dimethyl sulfoxide (DMSO).

**Figure 2:**
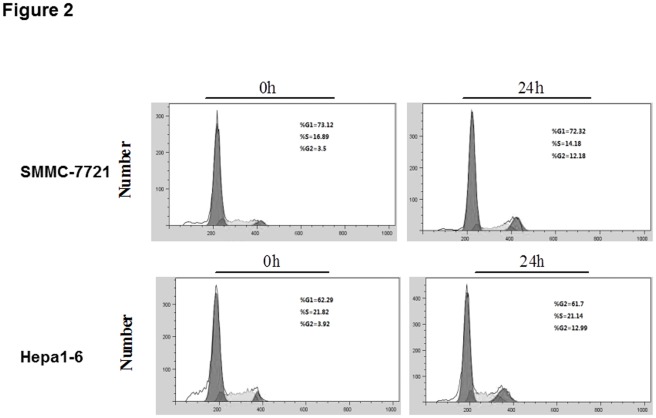
Effects of Osthole on Cell Cycle of HCC Cells. Cell cycle analysis of SMMC-7721 and Hepa1-6 cells following 123.0 µM osthole treatment for 24 h by flow cytometry.

**Figure 3:**
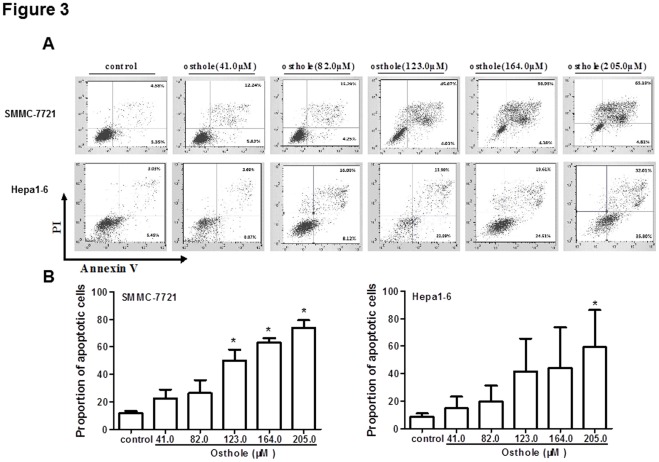
Effects of Osthole on apoptosis of HCC cells. (A) Induction of apoptosis of SMMC-7721 and Hepa1-6 cells after osthole treatment. SMMC-7721 and Hepa1-6 cells were treated with osthole at doses of 0, 41.0, 84.0, 123.0, 164.0 and 205.0 µM for 48 h. Apoptosis was measured by flow cytometry. (B) Statistical analysis of the percentages of the apoptotic cells. Data shown were representatives of three experiments.

### Cell Viability Assay

The effect of osthole on cell viability was measured by 3-(4,5-dimethylthiazol-2-yl)-2,5-diphenyl tetrazoliumbromide (MTT) assay. The cells were plated at a density of 2.5×10^3^ per well in 96-well plates overnight and then treated with different concentrations of osthole (0, 20.5, 41.0, 82.0, 123.0, 164.0 and 205.0 µM). The final concentrations of DMSO and ethanol were lower than 0.25%. After incubation for 24 h, 48 h and 72 h at 37°C in a humidified incubator, MTT (5 mg/ml in phosphate buffered saline (PBS)) was added to each well and incubated for 4 h; then the medium was totally removed, 0.1 ml of buffered DMSO was added to each well. The absorbance was recorded on a microplate reader (Sepctra Max M2e, Molecular Devices, Silicon Valley, CA) at the wavelength of 490 nm. The effect on cell proliferation was assessed as the percent cell viability wherein vehicle-treated cells were taken as 100% viable.

**Figure 4:**
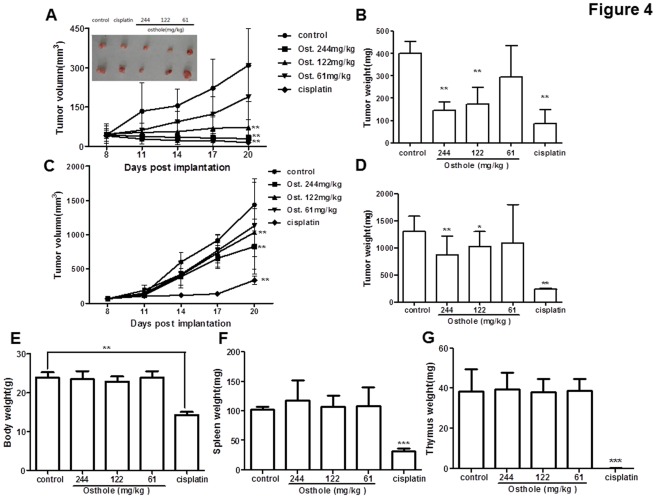
Effect of Osthole Treatment on the Tumorigenicity of HCC Cells. A total of 2×10^6^ SMMC-7721 or Hepa1-6 cells were inoculated subcutaneously (s.c.) into the right flank of nude mice or C57/BL6 mice. Mice were randomized into five groups including osthole treatment (244 mg/kg, 122 mg/kg and 61 mg/kg), corn oil alone as the blank control and cisplatin (5 mg/kg) as the positive control on Day8 and were treated once every other day for 2 weeks. (A) The tumor volumes of the nude mice inoculated with SMMC-7721 cells were measured and calculated once every three days. The tumor sizes on day 21 were shown in the inserted figure. (B) Tumor weights of the nude mice inoculated with SMMC-7721 cells were measured on day21. (C) The tumor volumes of the C57/BL6 mice inoculated with Hepa1-6 cells were measured and calculated once every three days. (D) Tumor weights of the C57/BL6 mice inoculated with Hepa1-6 cells were measured on day21. (E) The body weights of nude mice inoculated with SMMC-7721 cells were weighed on day 21. The (F) spleen weights and (G) thymus weights of the C57/BL6 mice inoculated with Hepa1-6 cells were weighed on day21. Each data point represented the mean±S.D. of 10 mice. Data shown were the representatives of three experiments.

### Cell Cycle Analysis

After osthole treatment, the DNA content and cell cycle distribution of SMMC-7721 and Hepa1-6 cells were determined by flow cytometry. Cells plated at a density of 5×10^5^ per well in 6-well plates were treated with osthole and harvested at 24 h. The cells were washed once in PBS. They were then fixed in cold 70% ethanol and stored at 4°C for 30 min. Then ethanol was removed and the cells were resuspended in PBS. The fixed cells were then washed with PBS, treated with RNase (100 µg/ml), and stained with Propidium Iodide (PI, 20 µg/ml) in the dark for 30 min at 37°C. Cell cycle was analyzed by flow cytometry (BD Biosciences, Franklin Lakes, NJ) and analyzed by Flowjo software.

**Figure 5:**
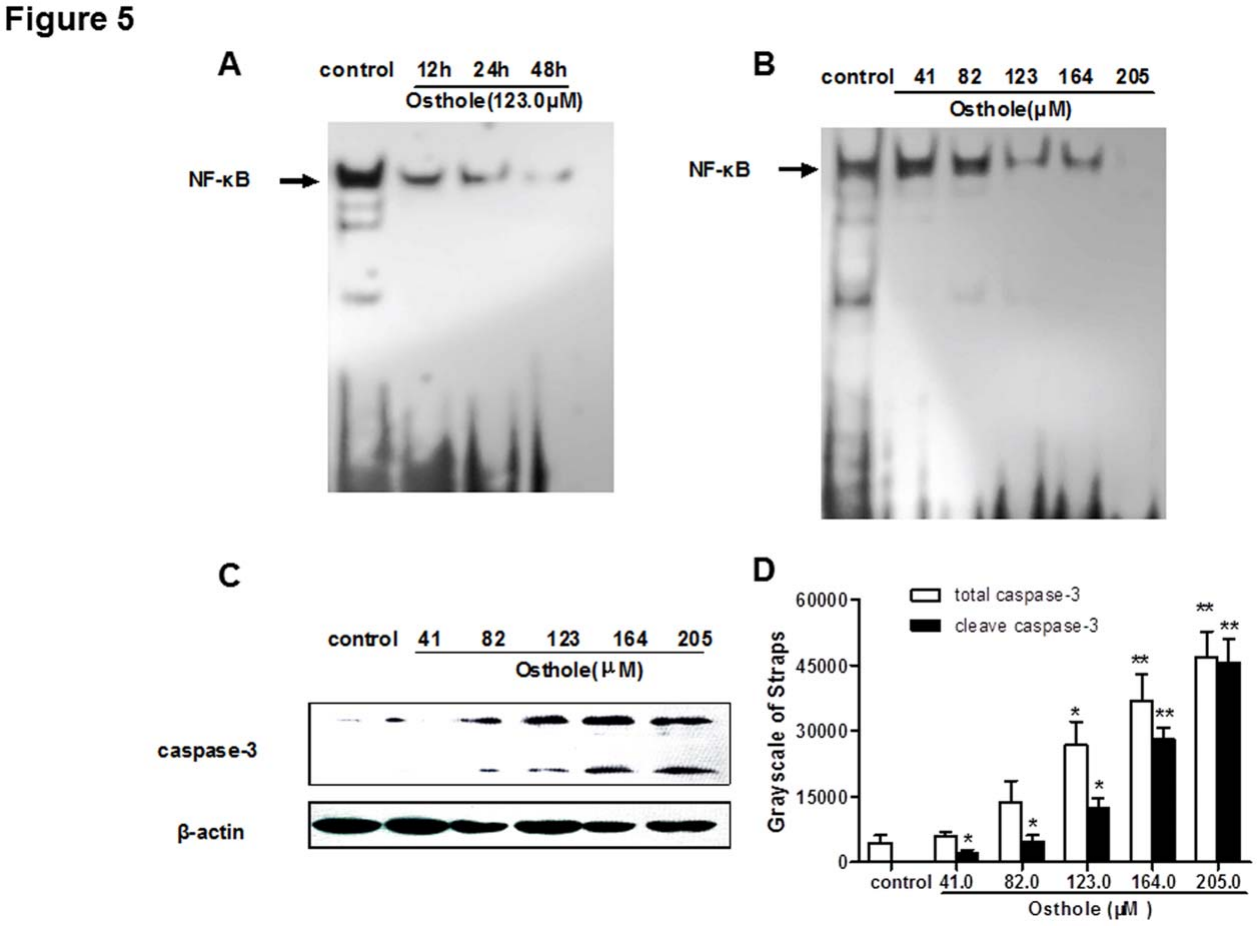
Effects of Osthole Treatment on Caspase-3 expression and NF-κB activation. (A) SMMC-7721 cells were treated with osthole (123.0 µM) for 12 h, 24 h and 48 h. The nuclear proteins were prepared and analyzed for NF-κB expression by EMSA. (B) SMMC-7721 cells were treated with osthole at doses of 0, 41.0, 82.0, 123.0, 164.0 and 205.0 µM for 24 h. The nuclear proteins were prepared and analyzed for NF-κB expression by EMSA. (C) SMMC-7721 cells were treated with osthole at doses of 0, 41.0, 82.0, 123.0, 164.0 and 205.0 µM for 48 h. The cell lysates were prepared and analyzed for caspase-3 expression by Western blot analysis. Equal loading was confirmed by stripping immunoblots and reprobing for β-actin. Data shown were representatives of three experiments. (D) Statistical analysis of caspase-3 quantification. * *p*<0.05, ** *p*<0.01.

### Quantification of Apoptosis

For apoptosis analysis, SMMC-7721 and Hepa1-6 cells (5×10^5^) were plated in each well of the 6-well plates and treated with different doses (0, 41.0, 82.0, 123.0, 164.0 and 205.0 µM) of osthole in 10% fetal bovine serum- DMEM for 48 h. The cells were then labeled with Annexin V and Propidium Iodide (PI) (Key Gene, JiangSu, China). Apoptotic rates were determined by flow cytometry (BD Biosciences, Franklin Lakes, NJ) and analyzed by Flowjo software. The percentage of the early apoptosis was calculated by Annexin V-positivity and PI-negativity, while the percentage of the late apoptosis was calculated by Annexin V-positivity and PI-positivity.

**Figure 6:**
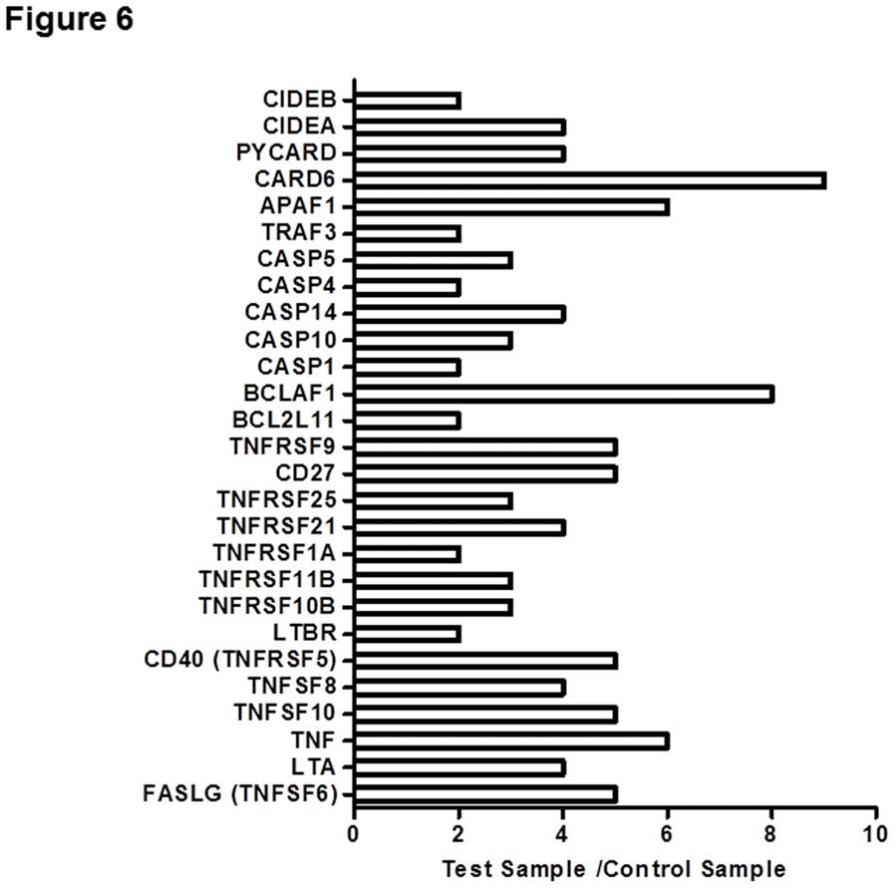
Effects of Osthole on Expressions of Apoptosis-Related Genes. SMMC-7721 cells were treated with osthole (123.0 µM) for 48 h. The expressions of apoptosis-related genes were analyzed using RT^2^ Profiler PCR Arrays. Increased expressions of two folds or more were shown. Data shown were representatives of two experiments.

### Animal Studies

A total of 2×10^6^ SMMC-7721 cells (or Hepa1-6 cells) suspended in 0.2 ml PBS were inoculated subcutaneously (s.c.) into the right flank of 50 nude mice (or C57/BL6 mice) using 1-ml needles. A week later, the mice were randomly distributed into five groups and each group consisted of 10 mice. They were treated with 244 mg/kg (1.0 mmol/kg), 122 mg/kg (0.5 mmol/kg) and 61 mg/kg (0.25 mmol/kg) osthole intraperitoneally (i.p.) in 0.2 ml corn oil, with corn oil alone as the control group and cisplatin as the positive control once every other day for 2 weeks. The tumor volumes were measured once every three days. The following formula was used for tumor volume measurement: (short diameter)^2^ * long diameter/2. On day 21, mice were sacrificed and tumor tissues, spleens and thymus were weighed. The Ethical Committee of Soochow University specifically approved the current study.

### Western Blot Analysis

SMMC-7721 cells were treated with osthole (0, 41.0, 82.0, 123.0, 164.0 and 205.0 µM) for 48 h in complete cell medium. After 48 h of treatment, cells were harvested and cell lysates were prepared and stored at -80°C for later use. The protein content in the lysates was determined using BCA Protein Assay Kit (Beyotime, Jiangsu, China). For Western blot analysis, 50 µg of protein from each sample were subjected to separate on a SDS-PAGE gel. After electrophoresis, proteins were electroblotted to polyvinylidene difluoride (PVDF) membranes, and subsequently incubated in blocking buffer (5% nonfat dry milk) for 12 h at 4°C. The blots were incubated with appropriate primary antibody, washed, and incubated with horseradish peroxidase (HRP)-conjugated secondary antibody (Dako, Carpinteria, CA). The blots were detected with chemiluminescence (ECL-Kit, Beyotime, JiangSu, China) followed by autoradiography. Equal loading of protein was confirmed by stripping the blots and reprobing with β-actin antibody.

### Electrophoretic Mobility Shift Assays (EMSA)

SMMC-7721 cells were plated at a density of 2×10^6^ per well in 6-well plates with or without osthole treatment and harvested at 12 h, 24 h and 48 h. Nuclear protein of SMMC-7721 cells were extracted using Nuclear and Cytoplasmic Protein Extraction Kit (Beyotime, Jiangsu, China) and protein content was determined using BCA Protein Assay Kit (Beyotime, Jiangsu, China). Nuclear protein (10 µg) was incubated with 10× binding buffer, 1.0 µg/µl poly(dI-dC), and 0.5 µl biotin-labeled double-stranded NF-κB binding consensus oligonucleotides 5′-AGTTGAGGGGACTTTCCCAGGC-3′(total volume 15 µl) in an EMSA Kit (Viagene Biotech, Tampa, FL). The binding reaction was performed for 25 min at room temperature. The incubation mixtures were separated by 6.5% non-denaturing PAGE and the bands were detected by autoradiography.

### Real-time PCR

SMMC-7721 cells were plated at a density of 5×10^5^ per well in 6-well plates with or without osthole (123.0 µM) treatment for 48 h. RNA was extracted from the cells using TRIzol reagent (Invitrogen, Carlsbad, CA). RNA concentration was determined by UV spectrophotometry (concentration by A260 should be greater than 40 µg/ml total RNA). RNA was converted to cDNAs. The real-time PCR was performed using an RT^2^ Profiler PCR Arrays (SABiosciences, Frederick, MD). Experimental cocktail was prepared by adding the first strand cDNAs synthesis reaction (102 µl) to 2X SABiosciences RT^2^ qPCR master mix (1350 µl) and water (1248 µl). Add 25 µl of the experimental cocktail to each well of the PCR Array. The plates were centrifuged for 1 minute at room temperature at 1000 g to remove bubbles. PCR were run with a Biosystems 7500 Fast Real- Time PCR System (Applied Biosystems, Foster, CA). The thermal cycle profile was as follows: 15 s at 95°C, 60 s at 60°C for 40 cycles. The data were calculated and analyzed by △△Ct.

### Statistical Analysis

All data represents at least three independent experiments and results were shown as mean±SD. Statistical differences between two groups were determined by Student’s *t*-test. A significant difference was considered as *p*<0.05.

## Results

### Effect of Osthole on Cell Viability and Apoptosis of HCC Cell Lines

Chemical structure of osthole was shown in figure 1A. In order to determine the effects of osthole on HCC, human HCC cell lines SK-HP-1, SMMC-7721, and HepG-2 and murine HCC cell line Hepa1-6 were treated with osthole at different doses for 48 h (Fig. 1B). Osthole treatment inhibited the proliferation of all four HCC cell lines in a dose-dependent manner. There was no significant difference in drug sensitivity (IC_50_: 189.5 µM, 161.9 µM, 161.4 µM and 137.0 µM, respectively) between the four HCC cell lines. Further experiments showed that osthole treatment inhibited the proliferation of SMMC-7721 and Hepa1-6 cell lines in a time-dependent manner (Fig. 1C&D).

The effect of osthole on cell cycle distribution was evaluated by flow cytometry. When osthole was administered at the dose of 123.0 µM, SMMC-7721 and Hepa1-6 cells both exhibited increased cell percentages in G2 phase (Fig. 2) with an increase of SMMC-7721 cells from 3.50% to 12.18% and Hepa1-6 cells from 3.92% to 12.99%. To further investigate whether Osthole could induce apoptosis of the HCC cells, the apoptotic cell percentages were analyzed by flow cytometry. SMMC-7721 and Hepa1-6 cells were treated with different concentrations of osthole (0, 41.0, 84.0, 123.0, 164.0 and 205.0 µM) for 48 h. The percentages of apoptotic cells were significantly increased in the treated group compared to control group (*p*<0.05) (Fig. 3A&B) for both cell lines in a dose-dependent manner. The apoptotic cells increased from total about 10% to 70% for SMMC-7721 cells and 60% for Hapa-1-6 cells. Taken together, osthole treatment could induce HCC cells apoptosis and G2 phase arrest.

### Osthole Suppressed the Tumor Growth in Murine Models of HCC

In order to determine the tumor suppressive effect of osthole *in vivo*, we next examined the effect of osthole in murine models of HCC. Nude mouse were subcutaneously inoculated with 2×10^6^ SMMC-7721 cells (day 1). Osthole treatment started on day8 and was administered at 244 mg/kg, 122 mg/kg and 61 mg/kg, intraperitoneally for 2 weeks with corn oil as the blank control and cisplatin (5 mg/kg) as the positive control. The data showed that tumor development was significantly suppressed in osthole (244 mg/kg and 122 mg/kg) -treated mice (29±14 mm^3^, 72±32 mm^3^) compared with the control group (310±139 mm^3^) (*p*<0.01) (Fig. 4A). The tumor weights of osthole (244 mg/kg and 122 mg/kg)-treated mice (144.3±40.1 mg, 174.0±72.8 mg) were significantly less than those of the control group (402.0±51.8 mg) (*p*<0.01) (Fig.4B).

Another murine model of HCC was established by subcutaneously inoculating 2×10^6^ Hepa1-6 cells in C57/BL6 mice. The mice were treated with osthole at 244 mg/kg, 122 mg/kg and 61 mg/kg intraperitoneally for 2 weeks starting on day 8. Compared with the control group, osthole (244 mg/kg and 122 mg/kg) treatment also significantly suppressed tumor growth (Fig. 4C) and reduced the tumor weights on day 21 (*p*<0.05, *p*<0.01) (Fig.4D). These results suggested that osthole was an effective agent that could inhibit the growth of transplanted HCC tumors *in vivo*.

There were no difference in body weight in three osthole treatment groups (23.50±2.06 g, 22.90±1.29 g, 23.90±1.64 g) in SMMC-7721 model compared with the control group (23.90±1.39 g), but the body weights in cisplatin group (14.30±0.76 g) were significantly decreased (*p*<0.01) (Fig. 4E). The Hepa1-6 HCC model showed similar results (data not shown). Moreover, the weights of spleens and thymus of three osthole treatment groups (spleens: 117.8±34.3 mg, 107.6.±17.8 mg, 108.8±30.8 mg; thymus: 39.5±8.3 mg, 38.0.±6.6 mg, 38.6±6.2 mg) showed no difference compared with those of the control group (spleens: 102.0±4.9 mg; thymus: 38.4±11.0 mg), while both spleen and thymus weights dropped dramatically in the cisplatin group (spleens: 32.0±4.2 mg, *p*<0.001; thymus: 0.04±0.1 mg, *p*<0.001, Fig. 4F&G). Therefore, osthole exhibited no apparent sign of toxicity in murine HCC models.

### Effects of Osthole on NF-κB Activity, Capase-3 and Other Apoptotic-related Gene Expressions

To further illustrate the molecular basis of the apoptosis induction by osthole, we investigated the effect of osthole on NF-κB activity. In SMMC-7721 cells, osthole significantly suppressed NF-κB activity in a time- (Fig. 5A) and dose- (Fig. 5B) dependent manner. The expression levels of caspase-3 after osthole treatment (0, 41.0, 82.0, 123.0, 164.0 and 205.0 µM) were also measured in SMMC-7721 cells. The total caspase-3 and the cleaved caspase-3 levels were both increased with osthole treatment in a dose-dependent manner (Fig. 5C).

To further demonstrate the effects of osthole on the apoptotic signaling pathways, we measured the expression levels of a set of apoptosis-relevant genes with or without osthole treatment in SMMC-7721 cells using RT^2^ Profiler PCR Arrays. The results showed that most apoptosis-related genes had increased expression levels with osthole treatment, which belonged to TNF ligand family, TNF receptor family, Bcl-2 family, caspase family, TRAF family, death domain family, CIDE domain and death effector domain family and CARD family (Fig. 6). Most changes were seen with the genes of the TNF ligand and receptor families. These results suggested that osthole treatment could induce apoptosis by up-regulating apoptosis-related gene expressions, especially TNF ligand and receptor gene expressions.

## Discussion

This is the first report on the anti-tumor effect of osthole in HCC. Our data demonstrated that osthole could inhibit the proliferation of HCC cell lines *in vitro* and suppress HCC tumor growth *in vivo*. It could down-regulate NF-κB activity and up-regulate most apoptosis-related genes. Our findings indicated that osthole could be developed as a novel anti-tumor agent for treating HCC.

Recently, it has been suggested that osthole could induce G2/M arrest and apoptosis in lung cancer A549 cells by modulating PI3K/Akt pathway [33]. Our results also suggested osthole could induce apoptosis and G2/M arrest in HCC cells. Meanwhile we demonstrated that osthole could inhibit NF-κB activation. It has been shown that the activated NF-κB could promote G2/M transition through inhibiting GADD45 expression following up-regulating Cyclin B [36]. Prostate cancer cells could be arrested in G2/M phase and undergo apoptosis by NF-κB inhibition [37]. Therefore, osthole could induce G2/M arrest and apoptosis through inhibiting NF-κB activity in HCC cells.

Multiple cellular receptors and signaling pathways are involved in promoting NF-κB activation, which plays a central role in HCC development, progression, and therapy [38]. It has been shown that the levels of NF-κB expression are higher in cancer tissues in HCC [39]. Various methods of HCC intervention, including proteasome inhibitor, IκB kinase (IKK) gene knockout, IκBα super-inhibitor, and interfering with NF-κB oligonucleotide or RNA, indicated that inhibiting the activity of NF-κB could inhibit the growth of HCC cells [40], [41], [42], [43]. It has been shown that thalidomide could effectively postpone or hinder the HCC development through inhibiting NF-κB activation in rat model [44]. Furthermore, one of the causes for apoptosis- and drug-resistance to occur in HCC cells was the activation of NF-κB [45]. Our results demonstrated that osthole could inhibit NF-κB activity in HCC cell lines in a dose- and time-dependent manner. Therefore, the proliferation inhibition in HCC cell lines by osthole could be due to its suppressive effect on NF-κB activity.

In nude mouse model of HCC, osthole administered at 244 mg/kg and 122 mg/kg significantly suppressed the growth of SMMC7721 cell-derived tumors and the similar tumor-suppressive effect was shown in C57/BL6 mice carrying murine Hepa1-6 cell-derived tumors. Interestingly, the lower dose of 61 mg/kg did not exhibit significant suppressive effects, which suggested that a relatively high dose of osthole was required to achieve therapeutic effects. This may also suggest that it could be the derivatives of osthole through the liver metabolism that exert the therapeutic effects on HCC. In both human and murine HCC models, the body weights of osthole treated groups were not different from those of the control group. In the immune competent murine HCC model, the spleen weights and thymus weights of osthole treated groups were also similar as those of the control group. Therefore, osthole exhibited no apparent signs of toxicity in murine HCC models without injuring immune organs. This also justified the safety of the high dose of osthole required for treating HCC. However, some coumarins may cause idiosyncratic hepatotoxicity and this requires further toxicity studies and should be taken into considerations during clinical trials.

Coumarins have been shown to undergo differential metabolisms in mice, rats and humans [46], [47]. The 7-hydroxylation pathway of coumarin metabolism is the major pathway in human but is only a minor pathway in the rat and mouse. It is a detoxification pathway and negatively correlated with the toxicity in the target organs, such as lung and liver. In contrast, the major route of coumarin metabolism in the rat and mouse is by a 3,4-epoxidation pathway resulting in the formation of toxic metabolites. The liver toxicity of coumarins could also lead to tumor formation in rats and female mice [48]. Therefore, the toxicity and carcinogenicity of coumarins are expected to be much lower in humans.

Although some coumarins have been shown to act as immune stimulants [49], osthole has been shown to have anti-inflammatory activities [25], [50]. It suppressed IL-4-induced eotaxin in BEAS-2B cells via inhibition of STAT6 expression and might have the potential for treating allergic airway inflammation [50]. Osthole could also attenuate the experimental autoimmune encephalomyelitis in C57 BL/6 mice and may be used for treating multiple sclerosis [51]. However, in osthole treated tumor-bearing immune competent mice, we observed no toxicity on immune organs and no significant decrease of peripheral or tumor infiltrating T cell numbers (data not shown). We hypothesize that osthole’s immune suppressive effect may only act on the activated immune cells. In tumor-bearing individuals, especially at the late stage of the disease, the immune system is normally suppressed. Therefore, the immune-suppressive effect of osthole may only play a minor role comparing to its anti-tumor effect during the treatment.

Real-time PCR results showed that osthole increased the mRNA levels of apoptosis-related genes belonged to TNF ligand family, TNF receptor family, Bcl-2 family, caspase family, TRAF family, death domain family, CIDE domain and death effector domain family and CARD family. One study reported that osthole induced apoptosis in A549 cells through down-regulating the expressions of Bcl-2 and up-regulating the expressions of Bax [33]. However, our data did not show significant changes in Bcl-2 and Bax expressions (data not shown). On the other hand, the expression levels of the genes belonged to TNF ligand and TNF receptor families were up-regulated by osthole treatment. However, the exact molecular targets and mechanisms of apoptosis induction by osthole treatment still need further investigations. Our results provided basis for mechanistic studies of osthole’s anti-tumor effects in HCC.

Taken together, our results suggested that osthole could effectively inhibit HCC tumor growth *in vitro* and *in vivo* with no sign of toxicity through, at least in part, suppressing NF-κB activation and induction of the apoptotic pathways. Therefore, our results support the notion that osthole could be developed as a potential agent for treating HCC.
